# Chromatin landscapes and genetic risk for juvenile idiopathic arthritis

**DOI:** 10.1186/s13075-017-1260-x

**Published:** 2017-03-14

**Authors:** Lisha Zhu, Kaiyu Jiang, Karstin Webber, Laiping Wong, Tao Liu, Yanmin Chen, James N. Jarvis

**Affiliations:** 1 Department of Biochemistry, Jacobs School of Medicine and Biomedical Sciences, University at Buffalo, Buffalo, NY USA; 2 Graduate Program in Biological Sciences, Jacobs School of Medicine and Biomedical Sciences, University at Buffalo, Buffalo, NY USA; 3 Department of Pediatrics, Jacobs School of Medicine and Biomedical Sciences, University at Buffalo, Buffalo, NY USA; 4 Genetics, Genomics, & Bioinformatics Program, Jacobs School of Medicine and Biomedical Sciences, University at Buffalo, Buffalo, NY USA

**Keywords:** JIA, Neutrophils, CD4 + T cells, SNP LD blocks, Functional elements, Epigenetic regulation

## Abstract

**Background:**

The transcriptomes of peripheral blood cells in children with juvenile idiopathic arthritis (JIA) have distinct transcriptional aberrations that suggest impairment of transcriptional regulation. To gain a better understanding of this phenomenon, we studied known JIA genetic risk loci, the majority of which are located in non-coding regions, where transcription is regulated and coordinated on a genome-wide basis. We examined human neutrophils and CD4 primary T cells to identify genes and functional elements located within those risk loci.

**Methods:**

We analyzed RNA sequencing (RNA-Seq) data, H3K27ac and H3K4me1 chromatin immunoprecipitation-sequencing (ChIP-Seq) data, and previously published chromatin interaction analysis by paired-end tag sequencing (ChIA-PET) data to characterize the chromatin landscapes within the known JIA-associated risk loci.

**Results:**

In both neutrophils and primary CD4+ T cells, the majority of the JIA-associated linkage disequilibrium (LD) blocks contained H3K27ac and/or H3K4me1 marks. These LD blocks were also binding sites for a small group of transcription factors, particularly in neutrophils. Furthermore, these regions showed abundant intronic and intergenic transcription in neutrophils. In neutrophils, none of the genes that were differentially expressed between untreated patients with JIA and healthy children were located within the JIA-risk LD blocks. In CD4+ T cells, multiple genes, including *HLA-DQA1*, *HLA-DQB2*, *TRAF1*, and *IRF1* were associated with the long-distance interacting regions within the LD regions as determined from ChIA-PET data.

**Conclusions:**

These findings suggest that genetic risk contributes to the aberrant transcriptional control observed in JIA. Furthermore, these findings demonstrate the challenges of identifying the actual causal variants within complex genomic/chromatin landscapes.

**Electronic supplementary material:**

The online version of this article (doi:10.1186/s13075-017-1260-x) contains supplementary material, which is available to authorized users.

## Background

One of the significant findings to emerge from translational studies of polyarticular juvenile idiopathic arthritis (JIA) has been the observation that peripheral blood cells in children with this disease have distinctly abnormal expression profiles. These abnormalities can be observed in neutrophils [1], peripheral blood mononuclear cells (PBMC) [2], and whole blood expression profiles [3], and do not correct even when children achieve remission [4]. In neutrophils, the transcriptional abnormalities are linked to perturbations in fundamental metabolic processes [5]. Furthermore, both hypervariable gene analyses [6] and contingency analyses [5] suggest that these transcriptional aberrations reflect impairment of the mechanisms through which transcription is regulated and coordinated on a genome-wide basis [7].

In addition to identifying transcriptional abnormalities, there has also been considerable progress in our ability to identify genetic risk in JIA [8, 9]. Furthermore, we are beginning to understand the contribution that genetic variance makes to the heterogeneity of clinical response to first-line agents such as methotrextate [10]. However, taking genetic risk information and integrating it into a coherent, testable hypothesis of disease pathogenesis has remained challenging. A recent genetic fine mapping study by Hinks et al. [11] illustrates that challenge. While these authors were able to identify multiple previously unknown regions of genetic risk for JIA using the Illumina Immunochip, the majority of the risk-associated variants were located within non-coding regions of the genome. In this regard, JIA resembles almost every other complex trait that has been studied using genome-wide association studies (GWAS) [12, 13], including immunologic/autoimmune diseases [14]. Thus, although it is common to identify genetic risk loci in terms of the closest protein-coding gene, we are learning that the genome is far more complex than previously imagined, and that the presence of a risk-conferring single nucleotide polymorphism (SNP) near a specific gene is not *prima facie* evidence that the causal variants have anything to do with that particular gene [15].

We have previously demonstrated how the field might begin to make sense of the wealth of genetic data that are being generated by GWAS and fine mapping studies [16], and how understanding genetic risk might provide additional insight into the transcriptional abnormalities seen in JIA. We recently demonstrated that the majority of the disease-associated SNPs identified in a genetic fine mapping study by Hinks et al. that are situated within the non-coding genome are located within linkage disequilibrium (LD) blocks that are enriched for H3K4me1/H3K27ac histone marks, epigenetic signatures associated with enhancer function, in both neutrophils and CD4+ T cells. Several of these same LD blocks contain non-coding RNAs that were identified on RNA sequencing (RNA-Seq) and verified by reverse transcriptase polymerase chain reaction (rtPCR) [16].

Our earlier paper focused entirely on the novel risk regions identified in the Hinks study [11]. In the current study, we examined additional regions of genetic risk as recently reviewed by Hersh et al. [8] and Herlin et al. [9]. We demonstrated how understanding the transcriptome and functional, non-coding genome allows us to better understand the nature of genetic risk in JIA and the well-described transcriptional aberrations. In the current paper, we examine the chromatin landscapes in both CD4+ T cells and neutrophils. The former are widely accepted as important mediators of the pathobiology of JIA [17], and the latter have become the subject of increasing interest from the standpoint of the role(s) in childhood-onset rheumatic diseases [18]. We used publically available genomic data and our own RNA-Seq data to gain mechanistic insights into JIA disease processes from genetic risk data.

## Methods

### Defining LD regions

SNPs used in this query are listed in Table 1 and were previously reviewed by Hersh et al. [8], Herlin et al. [9] and Hinks et al. [11]. We used an SNP Annotation And Proxy search (SNAP) database (http://www.broadinstitute.org/mpg/snap) [19] to define LD blocks based on the location of each SNP. In brief, we used the settings as follows: SNP dataset: 1000 Genome pilot 1 and HapMap 3 (release 2), *r* ^2^ threshhold: 0.9, Population Panel: CEU, Distance limit: 500. We selected the smallest number as our start location and the largest number as our stop location for each defined LD block.

**Table 1:** Histone marks in the SNP LD blocks in neutrophils and CD4 + T cells

LD region	SNP	Neutrophils		CD4 + T cells	
		H3K4me1 mark	H3K27ac mark	H3K4me1 mark	H3K27ac mark
chr1: 206940310 - 206947167	rs1800896	Y	N	Y	Y
chr1: 25197155 - 25203390	rs4648881	Y	N	Y	N
chr11: 36336263 - 36371757	rs4755450, rs7127214	N	N	N	N
chr12: 111884608 - 111932800	rs3184504	Y	Y	Y	Y
chr12: 112486818 - 112906415	rs17696736	Y	Y	Y	Y
chr13: 40299842 - 40368601	rs7993214, rs9532434	N	N	Y	N
chr13: 43056036 - 43066523	rs34132030	N	N	N	N
chr16: 11400900 - 11435990	rs66718203	Y	Y	Y	N
chr18: 12821903 - 12880206	rs7234029	Y	Y	Y	Y
chr2: 100806514 - 100837567	rs10194635, rs6740838, rs1160542	Y	N	Y	Y
chr2: 191900449 - 191935804	rs3821236	Y	Y	Y	Y
chr2: 191943742 - 191970120	rs7574865	N	N	Y	N
chr22: 24234493 - 24237862	rs755622	Y	Y	Y	Y
chr3: 119125202 - 119243934	rs4688013, rs4688011	Y	Y	Y	Y
chr3: 46253650 - 46350716	rs79893749	Y	Y	Y	Y
chr4: 123141054 - 123548068	rs17388568	Y	Y	Y	Y
chr6: 112359543 - 112448654	rs2280153	Y	Y	Y	Y
chr6: 137959235 - 138006504	rs6920220	N	N	Y	N
chr6: 31492453 - 31543031	rs1800629	N	Y	Y	Y
chr7: 22774437 - 22811384	rs6946509, rs7808122	N	N	N	N
chr7: 28152193 - 28243473	rs10280937, rs73300638	Y	Y	Y	Y
chr9: 123636121 - 123723351	rs10818488	Y	Y	Y	Y
chr9: 123640500 - 123706382	rs2900180	Y	Y	Y	Y

*SNP* single nucleotide polymorphism, *LD* linkage disequilibrium, *chr* chromosome, *Y* yes, *N* no

### Analysis of neutrophil RNA-Seq data

We queried neutrophil RNA-seq data from previously published RNA-seq studies [16, 20, 21] by uploading the data into the University of California Santa Cruz (UCSC) Genome Browser. The previously defined LD blocks were queried and visually inspected for signal representing intronic and intergenic transcripts as described in our previous paper [16]. We then selected two representative transcripts (one intronic and one intergenic) to confirm the presence of non-coding transcripts in these regions.

### Verification of non-coding RNA transcripts identified in neutrophils within the JIA-associated LD blocks

Complementary DNA was synthesized from total RNA using an iScript cDNA Synthesis kit (Bio-Rad). rt-PCR was performed using a HotStar Master PCR kit (Qiagen) with a Veriti thermocycler (Life Technologies) and included a control with no reverse transcription to exclude the possibility of an artifactual result due to contaminating genomic DNA. The temperature profile consisted of an initial step of 95 °C for 10 minutes, followed by 35 cycles of 95 °C for 30 seconds, 60 °C for 30 seconds and 72 °C for 30 seconds, and then a final step of 72 °C for 10 minutes. PCR products were resolved in 1.5% agarose gel. The nucleotide sequences of the primers were as follows: for ncTNFa (chr6: 31,544,379-31,544,485), 5′-CCTAATTCTGGGTTTGGGTTTGGG-3′ (forward) and 5′-CCTACTTTCACCTCCATCCATCCT-3′ (reverse); for ncSTAT4 (chr2:191,915,516-191,915,617), 5′- GTCTTGTGCAACTTCTTCCTTTC-3′ (forward) and 5′- ACCCTGTGACTGTTTGAGATTAC-3′ (reverse).

### ENCODE transcription factor binding site (TFBS) enrichment

ENCODE TFBS data were downloaded from UCSC Genome Browser ENCODE data (http://hgdownload.cse.ucsc.edu/goldenPath/hg19/encodeDCC/wgEncodeRegTfbsClustered/). Only the TFBS information annotated for blood cells was used. The whole genome was binned to 100 bp bins and intersected with H3K4me1 or H3K27ac peak regions, which were used as background. Fisher’s exact test was applied to test the significance of the enrichment of TFBS of each transcription factor within H3K4me1 or H3K27ac peaks in LD blocks compared to other regions with H3K4me1 or H3K27ac peaks across the whole genome. We set the cutoff of the false discovery rate (FDR) to 0.05 for all the analyses.

### Identification of H3K4me1/H327ac histone marks within LD regions

We used H3K4me1/H3K27ac ChIP-Seq data previously generated by our group from neutrophils of healthy adults [16] as our reference source for H3K4me1/H3K27ac genomic locations in neutrophils [GEO:GSE66896]. The H3K4me1/H3K27ac ChIP-Seq data reported on the Roadmap Epigenomics collection were used as our reference source for H3K4me1/H3K27ac genomic locations in CD4+ T cells [GEO:GSE198927]. The intersection of H3K4me1/H3K27ac peaks within LD regions was obtained using the bedtools intersect procedure [22] as described in our previous paper [16].

#### Identifying chromatin interactions in CD4+ T cells within JIA risk loci

Functional DNA elements such as enhancers do not always regulate the nearest gene. Indeed, in a recent study of chromatin conformation using HiC in T and B cell lines, Martin et al. [15] showed that >80% of long distance chromatin interactions occurred at distances >500 kb. We therefore queried an existing chromatin interaction analysis by paired-end tag sequencing (ChIA-PET) dataset for CD4+ T cells (GSE32677) [23] to determine chromatin interactions within the JIA-associated risk loci. This dataset used H3K4me2 as a marker for active enhancers, and thus provides a means of estimating those genes/gene networks that are regulated by specific enhancers or groups of enhancers. The chromosome coordinates for the observed 6520 long-distance chromatin interactions were converted from hg18 to hg19 using UCSC Genome browser LiftOver tool (http://www.genome.ucsc.edu/cgi-bin/hgLiftOver). For these analyses, we queried all known JIA-associated risk regions.

### Identifying molecular pathways of disease-associated SNPs

One means of using genetic risk data to provide mechanistic insights is to identify specific biological pathways encompassed by SNP-associated genes [24, 25]. However, there is a considerable breadth of opinion about what constitutes an SNP-associated gene, as the actual causal variants may be a considerable distance (in genomic terms) from the risk-associated SNP. We therefore used the method of Brodie et al. [26] to identify molecular pathways encompassed by JIA-associated SNPs across broad genomic distances. We queried regions 200 kb upstream and downstream of the JIA-associated SNPs, as outlined previously [24] to define genes and pathways associated with JIA risk SNPs.

## Results

Our previous work had queried new SNPs recently identified in a genetic fine mapping study published by Hinks et al. [4]. In the current study, we queried SNPs that were identified in reviews by Hersh et al. [8] and Herlin et al. [9] and those SNPs in Hinks et al. [11] that have not been analyzed in previous work and have established associations with JIA (Table 1). We used these SNPs to generate LD blocks using the SNAP database [19] and examined the functional genomic elements within these defined regions. We included SNPs located within non-coding regions of the genome (introns or intergenic areas), i.e., the majority of the JIA-associated SNPs.

### Verification of non-coding transcripts within neutrophils in JIA-associated LD blocks

**Fig. 1:**
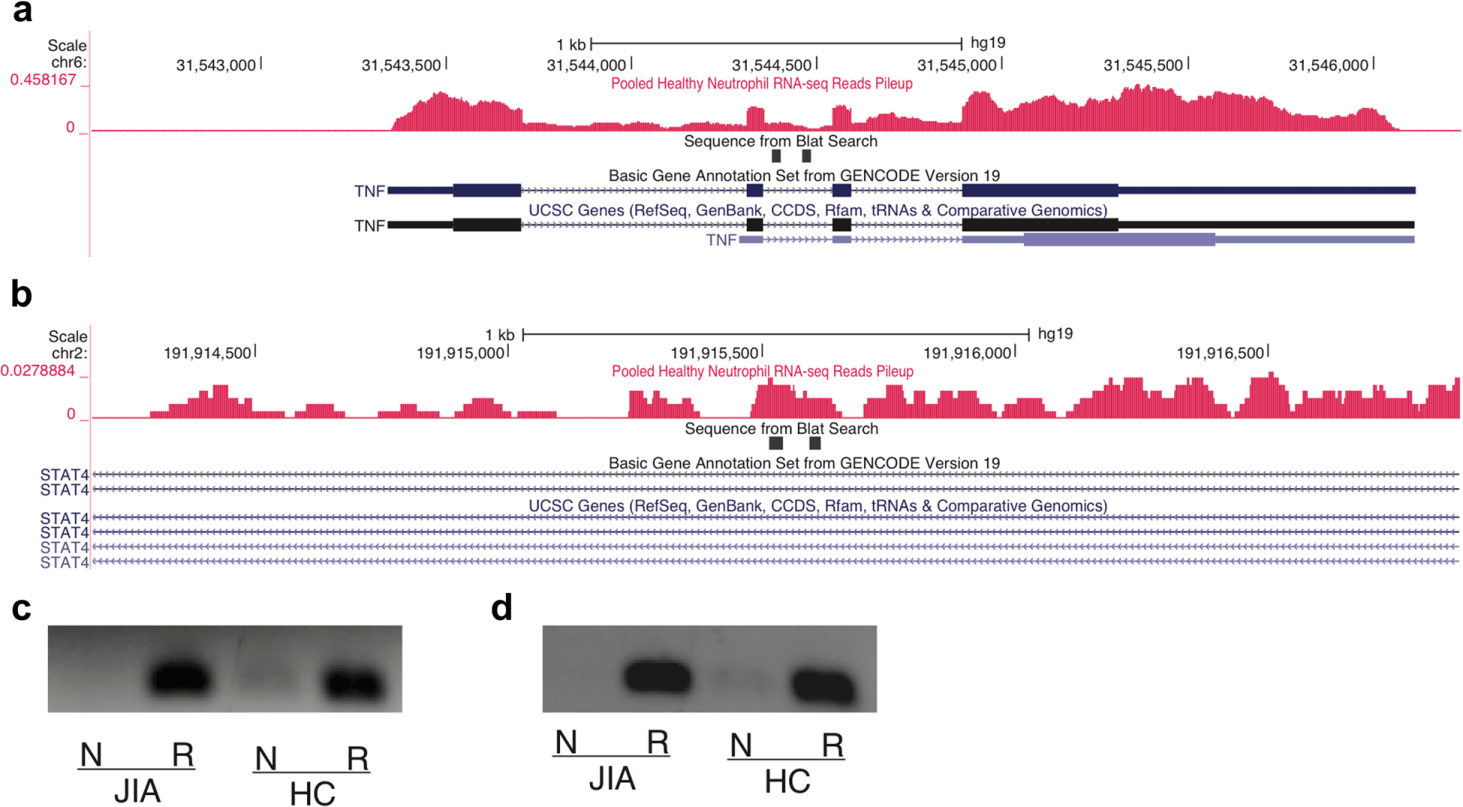
University of California Santa Cruz (*UCSC*) Genome Browser screen shot showing abundant intronic transcription in the *TNF* gene (**a**) and in the *STAT4* gene (**b**) within introns. *Black bars* indicate the region amplified by PCR experiments, as described in “Methods.” **c**, **d** Agarose gel images showing qualitative PCR amplification of intronic transcription (ncRNA) in the *TNF* gene (**c**) and in the *STAT4* gene (**d**) in neutrophils. *N* neutrophil RNA not subjected to reverse transcription, *R* neutrophil RNA subjected to reverse transcription prior to amplification. Neutrophils were isolated from patients with juvenile idiopathic arthritis (*JIA*) and healthy children (*HC*). Absence of signal in PCR amplifications that were performed without prior reverse transcription indicates that the signal was not being produced by contaminating DNA

Review of RNA-Seq data using the UCSC Genome Browser suggested the presence of multiple non-coding RNA transcripts in neutrophils within the JIA-associated LD blocks. We used rtPCR to confirm the presence of two of these transcripts, i.e., those within the LD blocks containing the rs3821236 and rs1800629 SNPs. Figure 1 shows detectable expression of these transcripts in neutrophils from a healthy child and a patient with JIA. This finding corroborates the idea that the additional JIA-associated LD blocks queried in the current paper contain functional, non-coding RNA transcripts, and corroborates the findings from our earlier paper [16].

### Location of H3K4me1/H3K27ac marks within LD blocks

For these analyses, we examined only those LD blocks not previously examined in our earlier paper [16] as noted in “Methods”. We identified LD blocks for all of the 30 risk SNPs, which were located in a total of 23 LD blocks. For those 23 LD blocks, we examined whether there are functional elements located in neutrophils or CD4+ T cells within these risk-conferring regions.

Our first analyses were in neutrophils. Of the 23 risk loci, 16 LD blocks contained H3K4me1 marks, while 14 LD blocks contained H3K27ac marks; 13 LD blocks contained both H3K4me1 and H3K27ac marks (Table 1). In all, 17 out of the 23 LD blocks contained either H3K4me1 or H3K27ac marks. The H3K4me1 and H3K27ac marks were significantly enriched in these LD block regions compared with non-LD block regions as determined by Fisher’s exact test (*p* value <2.2e-16 for both H3K4me1 and H3K27ac marks).

**Fig. 2:**
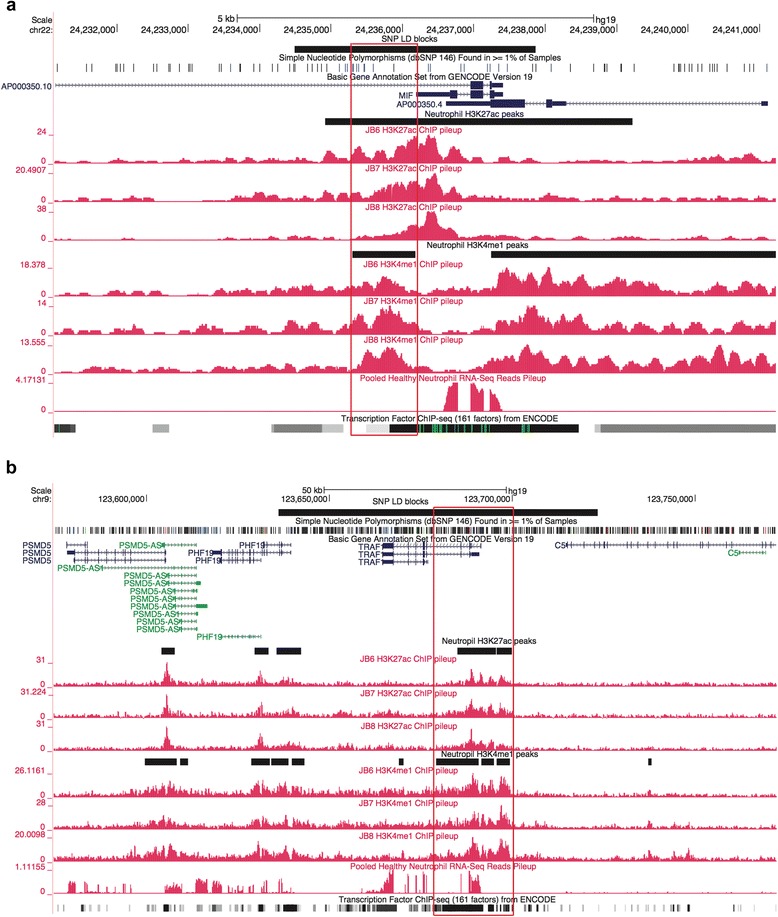
Representative screen shots from the University of California Santa Cruz Genome Browser show functional elements within neutrophil genomes. *Black horizontal bar* (*top*) represents the linkage disequilibrium (*LD*) blocks of the corresponding genome-wide association study single nucleotide polymorphism (*SNP*). *Black horizontal bars* between individual tracks represent H3K27ac and H3K4me1 peak regions generated from chromatin immunoprecipitation-sequencing (*ChIP-Seq*) data. *Gray bars* at the bottom of each image represent the transcription factor ChIP-seq of 161 factors from ENCODE with factorbook motifs. **a** LD block of rs755622 in neutrophils. Note there are expressed genes within this LD block, and the LD block contains H3K27ac and H3K4me1 regions with multiple potential transcription factor binding site (TFBS) within those regions. **b** LD block of rs2900180 in neutrophils. Note that there is one gene, the tumor necrosis factor receptor-associated factor (*TRAF1*), which is expressed within this LD block. This LD block also contains both H3K27ac and H3K4me1 peak regions with multiple TFBS within those regions. Potential active enhancers (region containing both H3K27ac and H3K4me1 peak regions) are highlighted in *red. MIF* macrophage inhibitory factor

Representative screen shots from the UCSC genome browser within two of the LD regions of interest are shown in Fig. 2. The LD block containing rs755622, for example (Fig. 2a), includes the promoter for the macrophage inhibitory factor (*MIF*) gene, but this region is also rich in transcription factor (TF) binding regions and contains H3K4me1/H3K27ac histone marks. Elevated MIF has been identified in patients with all three major JIA subtypes [27], although MIF is most elevated in children with the oligoarticular and systemic forms of the disease. Similarly, the neutrophil chromatin landscape around rs2900180 (Fig. 2b) has considerable genomic complexity. There is an expressed gene within this region, i.e., the TNF receptor-associated factor 1 (TRFA1)); there has been strong interest in the TNF signaling pathways since the efficacy of anti-TNF therapies was demonstrated in children with JIA [28]. At the same time, this region contains both H3K4me1/H3K27 marked enhancers and abundant TF binding not isolated to the TRAF promoter.

Similarly, Additional file 1: Figure S1 shows multiple functional elements adjacent to potentially disease-relevant genes such as PTPN2 (Additional file 1: Figure S1A) and NFkB inhibitor-like protein 1 (NFkBIL1, Additional file 1: Figure S1B). Thus, these findings corroborate our previous report [9] on the genetic risk regions identified in the Hinks paper [11], i.e., the genetic risk largely encompasses regions of the genome rich in functional elements that serve to regulate and coordinate transcription on a genome-wide basis.

We next examined H3K4me1/H3K27ac marked regions in CD4+ T cells in those same 23 LD blocks. We queried whether there are functional elements located in CD4+ T cells within these risk-conferring regions. Of the 23 LD blocks, 20 contained H3K4me1 marks and 15 contained H3K27ac marks. All of the 15 LD blocks that contained H3K27ac marks also contained H3K4me1 marks (Table 1). Thus, 20 out of 23 of the LD blocks contained either H3K27ac or H3K4me1 marks in CD4+ T cells. The H3K27ac and H3K4me1 marks are significantly enriched in these LD block regions compared with non-LD block regions as determined by Fisher’s exact test (*p* value <2.2e-16 for both H3K27ac and H3K4me1 marks).

### Transcription factor enrichment within the LD blocks

While H3K4me1/H3K27ac marks themselves do not unambiguously identify functional enhancers [29], the appearance of TFBS within H3K4me1/H3K27ac-marked regions is strong supportive evidence that these regions are functional [30]. Therefore, for each of the 38 LD blocks containing the 51 JIA-associated risk SNPs, we queried whether there was significant enrichment for TF binding in the H3K27ac/H3K4me1 marked regions. In these analyses, we included genetic risk information from the Hinks, Hersh, and Herlin papers. Thus, to determine whether there was additional evidence that the H3K4me1/H3K27ac marked regions within the JIA-associated LD blocks had functional significance, we queried whether there were significantly enriched TFBS within those loci compared to other genomic regions that have H3K4me1/H3K27ac marks.

**Fig. 3:**
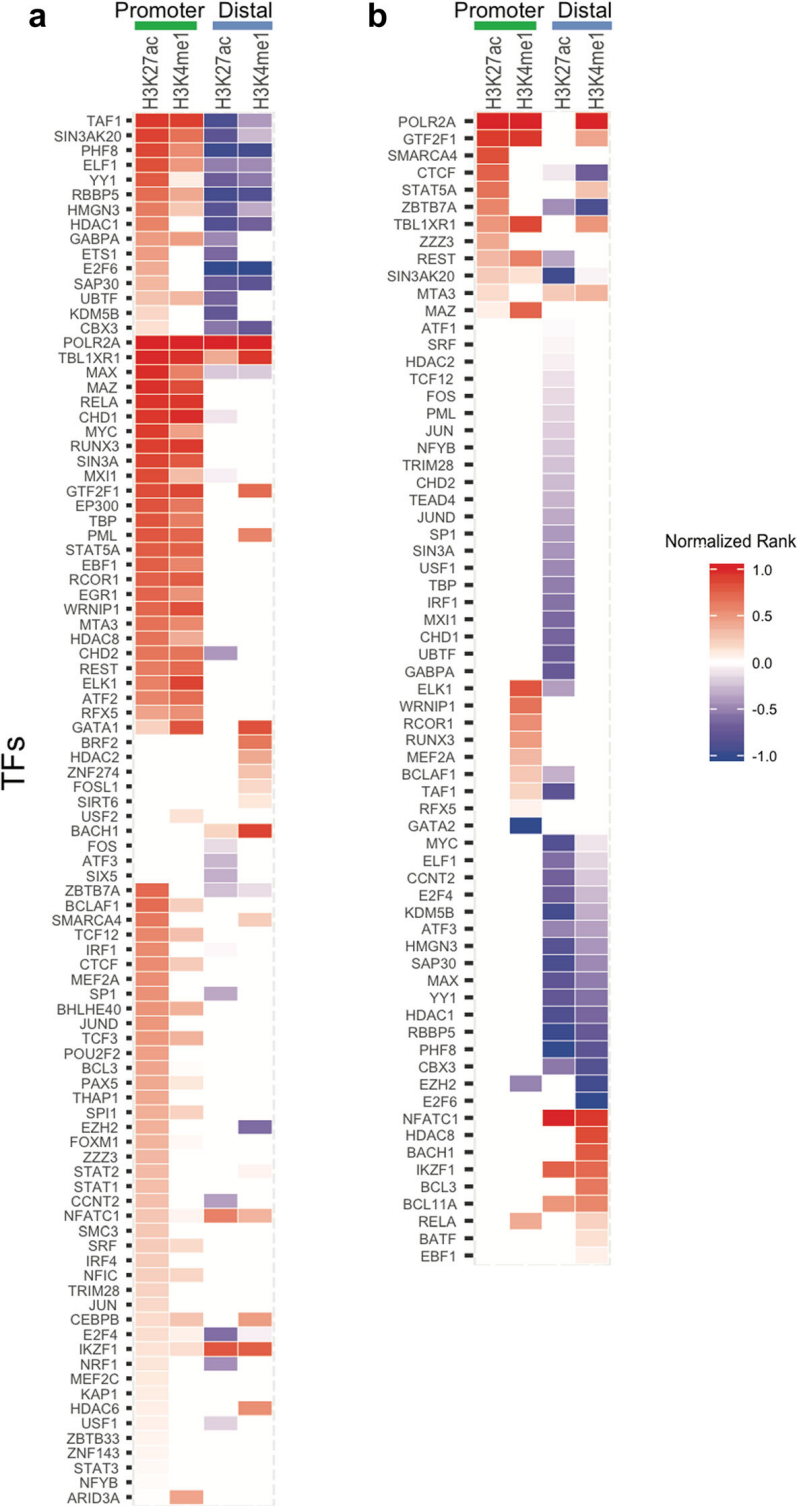
Enrichment of transcription factors (*TFs*) in H3K4me1/H3K27ac mark regions. Heatmap of significantly enriched TFs with their transcription factor binding sites (TFBS) in H3K27ac or H3K4me1 peak regions in neutrophil cells (**a**) and CD4 + T cells (**b**), considering promoter and distal regions separately. TFs are ranked according to false discovery rate (FDR) (the lowest FDR with the highest rank) and normalized to (0,1) by dividing the absolute highest rank value for enrichment (*red*) and depletion (*blue*) separately. (normalized rank >0 = enrichment, <0 = depletion)

For neutrophils, we observed highly enriched TF binding in promoter regions (defined as (-5 K, 1 K) of TSS) within these LD blocks. TFs that were particularly enriched included POLR2A, CHD1 and TAF1 (Fig. 3a). We noted that RUNX3 binding was also enriched within the H3K27ac-marked regions (Fig. 3a), an interesting finding in that the RUNX3 locus itself is associated with risk of JIA. This locus is characterized, in neutrophils, by dense intergenic TF binding, the presence of both H3K4me1 and H3K27ac peaks overlapping with the TF binding regions, and both intronic and intergenic RNA transcripts (not yet verified by rtPCR experiments; see Additional file 2: Figure S3). These findings suggest complex relationships between enhancers and promoters, and perhaps non-coding RNA transcripts, leading to complex levels of gene regulation in neutrophils in health and in disease states such as JIA.

For CD4+ T cells, we observed a limited number of TFBS that were significantly enriched in the LD blocks conferring risk of JIA that co-localized with H3K27ac/H3K4me1 histone marks (Fig. 3b). These regions were seen almost exclusively in promoters, defined as -5 K -1 K of the transcription start site (TSS). These transcription factors included POLR2A and GTF2F1. The majority of the distal (non-promoter) regions within these LD blocks were relatively depleted of TFBS. Thus, the LD blocks with H3K27ac/H3K4me1 marks are not highly enriched with TFs. This finding contrasts with what we observed in neutrophils, as noted above, where there is significant enrichment of TF binding within H3K4me1/H3K27ac-marked regions.

### Chromatin interactions in JIA risk loci

We used the 6520 long-distance chromatin interactions from a previously-reported ChIA-PET data analysis of CD4+ T cells [23] to define chromatin interactions in all the JIA-associated LD blocks. We identified 11 LD blocks that contained 48 long-range interactions. We noted that all six of the interaction regions within the LD block containing the SNP, rs10818488, are identical to the regions within the LD block of rs2900180. Thus, 42 unique long-range interactions remained, 11 of which displayed more than one interaction within or intersected with the same LD blocks (Additional file 3: Table S1). For these 42 long-range interactions, 20 were promoter-promoter (defined as (-5 K, 1 K) of TSS) interactions, 21 were promoter-distal (non-promoter) interactions, and one was a distal-distal interaction.

We next analyzed the ChIA-PET data in light of transcriptomes from CD4+ T cells derived from healthy children. We identified several genes within the CHIA-PET interacting regions, including ILB, DDX39B, RASSF5 and JAK1, which demonstrated high levels of expression (average fragments per kilobase of transcript (FPKM) >100). We noted that there are multiple small RNA molecules (snoRNA, miRNA, misc_RNA, snRNA) that are encoded within these regions, but the sequencing libraries were not prepared to detect their presence because the FPKM of those molecules is zero.

Among the 42 long-range interactions, 66 regions contained both H3K4me1 and H3K27ac marks, while 2 regions contained only H3K27ac marks and 11 contained only H3K4me1 marks; only 5 of the interacting regions contained neither H3K4me1 nor H3K27ac marks. There were in all 61 genes associated with these long-range interacting regions. The genes associated with regions containing both H3K4me1 and H3K27ac marks have average higher FPKM than those genes that contained only H3K4me1 marks or those with no histone marks (Additional file 4: Figure S2).

**Fig. 4:**
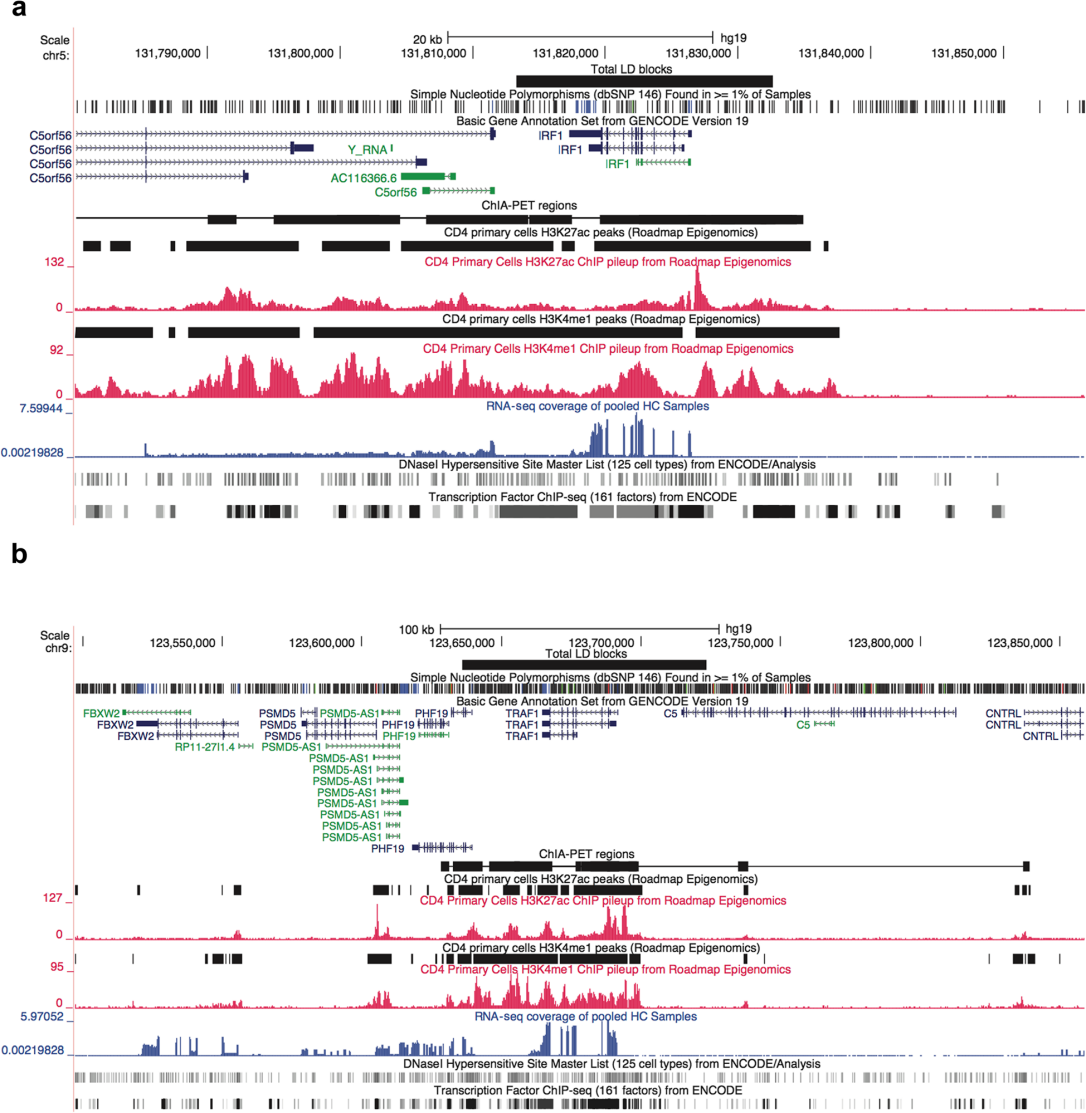
**a** Genome browser shot of the interferon regulatory factor 1 (*IRF1*)/C5orf56 locus in CD4 primary T cells, which is an epigenetically rich region showing dense transcription factor binding, multiple DNase1 hypersensitivity sites, and active enhancers with both H3K27ac and H3K4me1 marks. **b** TNF receptor-associated factor 1 (*TRAF1*) locus with rich epigenetic architecture in this locus, including multiple transcription factor binding sites, DNase1 hypersensitive sites, and active enhancers (H3K27ac and H3K4me1 marked regions). *CNTRL* control, *HC* healthy children

The ChIA-PET data point to complex layers of interaction and gene regulation within the JIA-associated risk loci. Note, for example, there are multiple points of interaction between the IRF1 and Corf56 genes, a locus that also encodes a Y-RNA species. These genes are physically adjacent to one another on chromosome 5, as shown in Fig. 4a. This region is dense with functional elements, including an H3K4me1/H3K27ac-marked enhancer, dense transcription factor binding, and multiple DNase1 hypersensitivity sites. It is interesting to note that our previous work from whole blood expression profiles of untreated patients with JIA suggested an important role for IRF1 networks in the pathogenesis of JIA [3]. Equally interesting is the physical interaction detected between the HLADQB1 and HLADQB2 loci. Association between JIA risk and class II HLA alleles has been long recognized, although the alleles at the DRB1 locus are thought to have stronger effects than those at the DQB1 or DQB2 loci [31]. The genome browser shot in Fig. 4b demonstrates the complexity of this locus. Complex interactions between TRAF1 and multiple adjacent genes (C5, PHF19, and CNTRL), and TRAF1 intragenic interactions were also observed on the ChIA-PET data by Chepelev et al. [23].

### Enriched molecular pathways of disease-associated SNPs

We used the method of Brodie et al. [24] and the PANTHER database [32] to identify significantly enriched biological processes and pathways for the 246 protein-coding genes and processed transcripts associated with the 51 JIA risk SNPs. Using a cutoff of a Bonferroni-adjusted *p* value <0.05, the enriched PANTHER pathways are related to interleukin signaling and inflammation mediated by chemokine and cytokine signaling. The enriched biological processes involve antigen processing and presentation, cellular defense response and other immunologically relevant processes (Additional file 5: Table S2).

## Discussion

In this paper, we mined existing datasets in order to gain a better understanding of the nature of genetic risk in JIA and therefore gain a better understanding of the transcriptional abnormalities that we and others have previously observed in untreated patients. We found that most of the JIA-associated SNPs lie within the non-coding genome, in regions that are dense with TF binding sites, DNaseI hypersensitive sites, epigenetic signatures of enhancer function, and, in the case of neutrophils, non-coding RNA transcripts. These findings strongly support the idea that genetic risk in JIA is determined by aberrant regulation of gene expression rather than the improper function of proteins mediated by an improper amino acid substitution. This idea is now gaining broad acceptance in the field of autoimmunity [33], and may provide the basis for understanding the transcriptional abnormalities seen in a broad spectrum of peripheral blood cells in JIA. These findings also demonstrate the importance of considering the chromatin context around disease-associated SNPs, which may have no functional association at all with the “nearest gene.”

At the completion of the Human Genome Project, there was some surprise and even a little dismay at how little of the genome contains protein-encoding genes. When the entire project was completed, only approximately 2% of the DNA was found to encode proteins [34]. One of the transformative findings from the ENCODE and Roadmap Epigenomics projects has been the discovery of the richness of the non-coding genome. Enhancers, insulators, CCCTC binding factor (CTCF) binding sites (which regulate chromatin conformation), etc., are broadly distributed throughout the genome and demonstrate that the processes of regulating gene expression and coordinating expression on a genome-wide basis is extraordinarily complex [35]. The field of immunology is now making considerable progress in identifying these functional elements and how they regulate transcription and therefore immune responses [36].

The findings from ENCODE and Roadmap Epigenomics, and the work we describe here, demonstrate that identifying specific causal variants in JIA is going to be an ongoing challenge. A primary reason for this is the complexity of the risk loci. While many of these loci encompass genes that are of strong immunologic interest (e.g., MIF, TRAF1, PTNP2, NFkBIL1), these same loci are also rich in other functional elements that regulate transcription on a global basis. For example, Fig. 2a is a simplified genome browser screen shot that shows the neutrophil chromatin landscape around rs755622. While the actual SNP is within the promoter of the MIF gene, the entire LD block contains both H3K4me1 and H3K27ac histone marks (strong evidence for an active enhancer in this region) and rich TF binding that is not localized exclusively to the promoter. Similar genomic features can also be seen in this region in CD4+ T cells (data not shown).

We also found a large degree of complexity around rs2900180 (Fig. 2b), and rs2847293/rs7234029, which are in the same LD block. The region containing rs2847293/rs7234029 is of broad general interest to the field of immunology, as this LD block includes the *PTPN2* gene. While PTPN2 is generally considered a regulator of T cell responses, this protein tyrosine phosphatase is broadly expressed in innate immune cells, including in neutrophils, where it serves as a negative regulator of cytokine signaling [37]. However, the PTPN2 locus is also characterized by H3K4me1/H3K27ac-marked enhancers in neutrophils and CD4+ T cells, and an apparent non-coding RNA molecule in the intron between the first and second exons in neutrophils (Additional file 1: Figure S1A). Thus, while it is tempting to focus on the protein-encoding genes within these regions as the location of the causal variants that lead to perturbed immune function, our data invite other interpretations. Enhancers, for example, may function at considerable distances (in genomic terms) from the genes they regulate, and often do not regulate the closest gene [38, 39]. This idea is shown in the recent work of Martin et al. [15]. Using Hi-C chromatin conformation capture, these authors demonstrated that >80% of long-distance chromatin interactions within T and B cell lines occurred at distances >500 Mb. Thus, until there is more solid experimental evidence to indicate otherwise, we must be open to the possibility that it is the enhancer function of these regions and/or the function of the ncRNA that are perturbed by genetic variance rather than specific functions or regulation of the nearby proteins.

This idea is corroborated by our analysis of the published CD4+ T cells ChIA-PET dataset. ChIA-PET uses crosslinked DNA to identify physical interactions between regions of DNA, which may either be in close proximity or many Mb apart on a specific chromosome (or even regions that are on different chromosomes) [23, 40]. The dataset we queried used H3K4me2 as a general mark for active enhancers. We found that the JIA-associated genetic risk loci display abundant and complex physical interactions with (usually) nearby genomic elements. The region encoding the TRAF1 gene is emblematic of this complexity. We detected interactions between an H3K4me2-marked region adjacent to the TRAF1 gene and at least three other genes. These genes included C5, the 5th component of the complement cascade, an important inflammatory mediator. The region also interacts with PHF19, which is itself a regulator of gene expression. PHF19 is a member of the polycomb repressive complex-2 (PRC-2) that is involved in gene silencing [41]. The significance of the interaction between the TRAF1 region and CNTRL (formerly for as CEP110), a centrosomic protein [42] is not immediately clear.

ENCODE also revealed that much of the genome is transcribed, and that many (if not most) of these non-coding RNA species are functional. In rheumatology, miRNA are the best-known of these non-coding RNA families, as numerous papers have shown links between specific miRNA molecules and specific immunologic features of the rheumatic diseases (e.g., [43]). Our own work has recently shown extensive re-wiring of mRNA-miRNA networks within JIA neutrophils [44]. However, the non-coding RNA world is broad, and includes RNA species that regulate chromatin accessibility [45], perform enhancer functions [46–48], and encode for small peptides [49]. Our current work also demonstrates the presence of at least two intronic, non-coding RNA molecules in the JIA-associated genetic risk loci. Such transcripts are abundant in neutrophils [50] and may regulate the decay of otherwise unstable mRNA transcripts [47]. The specific roles of these transcripts in neutrophil biology and/or the pathogenesis of JIA remain to be clarified. Their presence, however, serves to demonstrate that the localization of a disease-associated polymorphism within a specific gene does not necessarily mean that genetic risk is conferred by alterations in the protein-coding function of that gene.

The findings we report here provide an explanation for an observation that we have recently reported: differentially expressed genes in JIA are not situated within the LD blocks that confer genetic risk [3]. Using whole blood gene expression data from subjects of a National Institutes of Health-funded clinical trial, corroborated by an independent patient cohort, we reported that among >150 genes that were differentially expressed in patients and healthy controls, none were encoded within the LD blocks associated with JIA risk. Thus, the mechanisms through which genetic variance leads to perturbed gene expression are likely to be more complex than was previously considered. While it is possible that a different result might be obtained using RNA-Seq data from purified cell populations, the large number of gene-regulatory elements in the risk loci also invites us to consider that much of the genetic risk may be mediated by perturbation of long-range interactions, as suggested by the data reported by Martin et al. [15].

This paper adds to the mounting evidence that suggests that the field of pediatric rheumatology may require a fundamental re-assessment of theories on the pathogenesis of JIA. Long accepted as an “autoimmune disease” triggered by the recognition of a “self” peptide by the adaptive immune system, new evidence from the fields of genetics and genomics ought to prompt a reconsideration of this view. We propose, instead, that JIA emerges because of genetic and epigenetically mediated alterations in leukocyte genomes. These alterations, we propose, involve both innate and adaptive immune systems and lead to expression of inflammatory mediators in the absence of the external signals normally required to initiate or sustain an inflammatory response. Our work in both whole blood buffy coats [6] and neutrophils [5] is consistent with that interpretation of the available gene expression and genetic data.

## Conclusions

We demonstrated that the known genetic risk loci for JIA are enriched for multiple functional elements within the non-coding genome of human neutrophils. These functional elements include rich TF binding sites, H3K4me1 and H3K27ac-marked enhancers, and non-coding RNAs. We believe that these data and newer understanding of genetic risk and genome function invite new understanding of JIA pathogenesis, and may provide the basis for radical new approaches to therapy.

## Additional files

Additional file 1: Figure S1. Genome browser screen shots. Chromatin organization around PTPN2 and NFKBIL2 loci. (TIFF 20832 kb)
Additional file 2: Figure S3. Genome browser screen shots. Chromatin organization around the RUNX3 locus. (TIFF 10898 kb)
Additional file 3: Table S1. Summary of ChIA-PET peak regions intersecting with JIA-associated LD blocks. (XLSX 68 kb)
Additional file 4: Figure S2. Genes associated with these long-range interacting regions from CD4+ T cell ChIA-PET data. The genes associated with regions containing both H3K4me1 and H3K27ac marks have average higher FPKM than those genes that contained only H3K4me1 marks or those with no histone marks. (TIFF 17228 kb)
Additional file 5: Table S2. Enriched biological functions of genes within JIA-associated LD blocks. (XLS 28 kb)

## Abbreviations

bp: Base pair(s)
ChIA-PET: Chromatin interaction analysis by paired-end tag sequencing
ChIPseq: Chromatin immunoprecipitation-sequencing
FDR: False discovery rate
FPKM: fragments per kilobase of transcript
GWAS: Genome-wide association studies
JIA: Juvenile idiopathic arthritis
LD: Linkage disequilibrium
MIF: Macrophage inhibitory factor
ncRNA: Non-coding RNA
rtPCR: reverse transcriptase polymerase chain reaction
SNP: Single nucleotide polymorphism
SNAP: SNP Annotation And Proxy search
TF: Transcription factor
TFBS: Transcription factor binding site
TNF: tumor necrosis factor
TRAF: tumor necrosis factor receptor-associated factor
TSS: transcription start site
UCSC: University of California Santa Cruz

## References

[1] Jarvis JN, Jiang K, Frank MB, Knowlton N, Aggarwal A, Wallace CA, McKee R, Chaser B, Tung C, Smith LB (2009). Gene expression profiling in neutrophils from children with polyarticular juvenile idiopathic arthritis. Arthritis Rheum.

[2] Knowlton N, Jiang K, Frank MB, Aggarwal A, Wallace C, McKee R, Chaser B, Tung C, Smith L, Chen Y (2009). The meaning of clinical remission in polyarticular juvenile idiopathic arthritis: gene expression profiling in peripheral blood mononuclear cells identifies distinct disease states. Arthritis Rheum.

[3] Jiang K, Wong L, Sawle AD, Frank MB, Chen Y, Wallace CA, Jarvis JN (2016). Whole blood expression profiling from the TREAT trial: insights for the pathogenesis of polyarticular juvenile idiopathic arthritis. Arthritis Res Ther.

[4] Jiang K, Frank M, Chen Y, Osban J, Jarvis JN (2013). Genomic characterization of remission in juvenile idiopathic arthritis. Arthritis Res Ther.

[5] Jarvis JN, Petty HR, Tang Y, Frank MB, Tessier PA, Dozmorov I, Jiang K, Kindzelski A, Chen Y, Cadwell C (2006). Evidence for chronic, peripheral activation of neutrophils in polyarticular juvenile rheumatoid arthritis. Arthritis Res Ther.

[6] Dozmorov I, Knowlton N, Tang Y, Shields A, Pathipvanich P, Jarvis JN, Centola M (2004). Hypervariable genes–experimental error or hidden dynamics. Nucleic Acids Res.

[7] Komili S, Silver PA (2008). Coupling and coordination in gene expression processes: a systems biology view. Nat Rev Genet.

[8] Hersh AO, Prahalad S (2015). Immunogenetics of juvenile idiopathic arthritis: A comprehensive review. J Autoimmun.

[9] Herlin MP, Petersen MB, Herlin T (2014). Update on genetic susceptibility and pathogenesis in juvenile idiopathic arthritis.EMJ. Rheumatol.

[10] Moncrieffe H, Hinks A, Ursu S, Kassoumeri L, Etheridge A, Hubank M, Martin P, Weiler T, Glass DN, Thompson SD (2010). Generation of novel pharmacogenomic candidates in response to methotrexate in juvenile idiopathic arthritis: correlation between gene expression and genotype. Pharmacogenet Genomics.

[11] Hinks A, Cobb J, Marion MC, Prahalad S, Sudman M, Bowes J, Martin P, Comeau ME, Sajuthi S, Andrews R (2013). Dense genotyping of immune-related disease regions identifies 14 new susceptibility loci for juvenile idiopathic arthritis. Nat Genet.

[12] Maurano MT, Humbert R, Rynes E, Thurman RE, Haugen E, Wang H, Reynolds AP, Sandstrom R, Qu H, Brody J (2012). Systematic localization of common disease-associated variation in regulatory DNA. Science.

[13] Schaub MA, Boyle AP, Kundaje A, Batzoglou S, Snyder M (2012). Linking disease associations with regulatory information in the human genome. Genome Res.

[14] Farh KK, Marson A, Zhu J, Kleinewietfeld M, Housley WJ, Beik S, Shoresh N, Whitton H, Ryan RJ, Shishkin AA (2015). Genetic and epigenetic fine mapping of causal autoimmune disease variants. Nature.

[15] Martin P, McGovern A, Orozco G, Duffus K, Yarwood A, Schoenfelder S, Cooper NJ, Barton A, Wallace C, Fraser P (2015). Capture Hi-C reveals novel candidate genes and complex long-range interactions with related autoimmune risk loci. Nat Commun.

[16] Jiang K, Zhu L, Buck MJ, Chen Y, Carrier B, Liu T, Jarvis JN (2015). Disease-associated single-nucleotide polymorphisms from noncoding regions in juvenile idiopathic arthritis are located within or adjacent to functional genomic elements of human neutrophils and CD4+ T cells. Arthritis Rheumatol.

[17] Grom AA, Hirsch R (2000). T-cell and T-cell receptor abnormalities in the immunopathogenesis of juvenile rheumatoid arthritis. Curr Opin Rheumatol.

[18] Huttenlocher A, Smith JA (2015). Neutrophils in pediatric autoimmune disease. Curr Opin Rheumatol.

[19] Johnson AD, Handsaker RE, Pulit SL, Nizzari MM, O'Donnell CJ, de Bakker PI (2008). SNAP: a web-based tool for identification and annotation of proxy SNPs using HapMap. Bioinformatics.

[20] Hui-Yuen JS, Zhu L, Wong LP, Jiang K, Chen Y, Liu T, Jarvis JN (2016). Chromatin landscapes and genetic risk in systemic lupus. Arthritis Res Ther.

[21] Jiang K, Sun X, Chen Y, Shen Y, Jarvis JN (2015). RNA sequencing from human neutrophils reveals distinct transcriptional differences associated with chronic inflammatory states. BMC Med Genomics.

[22] Quinlan AR, Hall IM (2010). BEDTools: a flexible suite of utilities for comparing genomic features. Bioinformatics.

[23] Chepelev I, Wei G, Wangsa D, Tang Q, Zhao K (2012). Characterization of genome-wide enhancer-promoter interactions reveals co-expression of interacting genes and modes of higher order chromatin organization. Cell Res.

[24] Wang K, Li M, Hakonarson H (2010). Analysing biological pathways in genome-wide association studies. Nat Rev Genet.

[25] Brodie A, Tovia-Brodie O, Ofran Y (2014). Large scale analysis of phenotype-pathway relationships based on GWAS results. PLoS One.

[26] Brodie A, Azaria JR, Ofran Y (2016). How far from the SNP may the causative genes be?. Nucleic Acids Res.

[27] Meazza C, Travaglino P, Pignatti P, Magni-Manzoni S, Ravelli A, Martini A, De Benedetti F (2002). Macrophage migration inhibitory factor in patients with juvenile idiopathic arthritis. Arthritis Rheum.

[28] Lovell DJ, Giannini EH, Reiff A, Cawkwell GD, Silverman ED, Nocton JJ, Stein LD, Gedalia A, Ilowite NT, Wallace CA (2000). Etanercept in children with polyarticular juvenile rheumatoid arthritis. Pediatric Rheumatology Collaborative Study Group. N Engl J Med.

[29] Creyghton MP, Cheng AW, Welstead GG, Kooistra T, Carey BW, Steine EJ, Hanna J, Lodato MA, Frampton GM, Sharp PA (2010). Histone H3K27ac separates active from poised enhancers and predicts developmental state. Proc Natl Acad Sci U S A.

[30] Alexander RP, Fang G, Rozowsky J, Snyder M, Gerstein MB (2010). Annotating non-coding regions of the genome. Nat Rev Genet.

[31] Hollenbach JA, Thompson SD, Bugawan TL, Ryan M, Sudman M, Marion M, Langefeld CD, Thomson G, Erlich HA, Glass DN (2010). Juvenile idiopathic arthritis and HLA class I and class II interactions and age-at-onset effects. Arthritis Rheum.

[32] Mi H, Muruganujan A, Casagrande JT, Thomas PD (2013). Large-scale gene function analysis with the PANTHER classification system. Nat Protoc.

[33] Michailidou K, Beesley J, Lindstrom S, Canisius S, Dennis J, Lush MJ, Maranian MJ, Bolla MK, Wang Q, Shah M (2015). Genome-wide association analysis of more than 120,000 individuals identifies 15 new susceptibility loci for breast cancer. Nat Genet.

[34] The ENCODE Project Consortium. An integrated encyclopedia of DNA elements in the human genome. Nature, 2012;489:57–74. 10.1038/nature11247 PMC3439153 22955616

[35] Gandin V, Sikstrom K, Alain T, Morita M, McLaughlan S, Larsson O, Topisirovic I. Polysome fractionation and analysis of mammalian translatomes on a genome-wide scale. J Vis Exp. 2014;17. doi:10.3791/51455. 10.3791/51455 PMC4189431 24893926

[36] Winter DR, Jung S, Amit I (2015). Making the case for chromatin profiling: a new tool to investigate the immune-regulatory landscape. Nat Rev Immunol.

[37] Spalinger MR, McCole DF, Rogler G, Scharl M (2015). Role of protein tyrosine phosphatases in regulating the immune system: implications for chronic intestinal inflammation. Inflamm Bowel Dis.

[38] Shlyueva D, Stampfel G, Stark A (2014). Transcriptional enhancers: from properties to genome-wide predictions. Nat Rev Genet.

[39] Levine M, Cattoglio C, Tjian R (2014). Looping back to leap forward: transcription enters a new era. Cell.

[40] Li G, Cai L, Chang H, Hong P, Zhou Q, Kulakova EV, Kolchanov NA, Ruan Y (2014). Chromatin interaction analysis with paired-end tag (ChIA-PET) sequencing technology and application. BMC Genomics.

[41] Brien GL, Gambero G, O'Connell DJ, Jerman E, Turner SA, Egan CM, Dunne EJ, Jurgens MC, Wynne K, Piao L (2012). Polycomb PHF19 binds H3K36me3 and recruits PRC2 and demethylase NO66 to embryonic stem cell genes during differentiation. Nat Struct Mol Biol.

[42] Ou YY, Mack GJ, Zhang M, Rattner JB (2002). CEP110 and ninein are located in a specific domain of the centrosome associated with centrosome maturation. J Cell Sci.

[43] Liang D, Shen N (2012). MicroRNA involvement in lupus: the beginning of a new tale. Curr Opin Rheumatol.

[44] Hu Z, Jiang K, Frank MB, Chen Y, Jarvis JN (2016). Complexity and specificity of the neutrophil transcriptomes in juvenile idiopathic arthritis. Sci Rep.

[45] Li LC (2014). Chromatin remodeling by the small RNA machinery in mammalian cells. Epigenetics.

[46] Birnbaum RY, Clowney EJ, Agamy O, Kim MJ, Zhao J, Yamanaka T, Pappalardo Z, Clarke SL, Wenger AM, Nguyen L (2012). Coding exons function as tissue-specific enhancers of nearby genes. Genome Res.

[47] Li W, Notani D, Rosenfeld MG (2016). Enhancers as non-coding RNA transcription units: recent insights and future perspectives. Nat Rev Genet.

[48] Ahituv N (2016). Exonic enhancers: proceed with caution in exome and genome sequencing studies. Genome Med.

[49] Pauli A, Rinn JL, Schier AF (2011). Non-coding RNAs as regulators of embryogenesis. Nat Rev Genet.

[50] Wong JJ, Ritchie W, Ebner OA, Selbach M, Wong JW, Huang Y, Gao D, Pinello N, Gonzalez M, Baidya K (2013). Orchestrated intron retention regulates normal granulocyte differentiation. Cell.

