# Supplementary figures and images for: Chromatin landscapes and genetic risk for juvenile idiopathic arthritis
Source: Arthritis Res Ther. 2017 Mar 14;19:57. doi: 10.1186/s13075-017-1260-x (PMC5348874; doi:10.1186/s13075-017-1260-x)

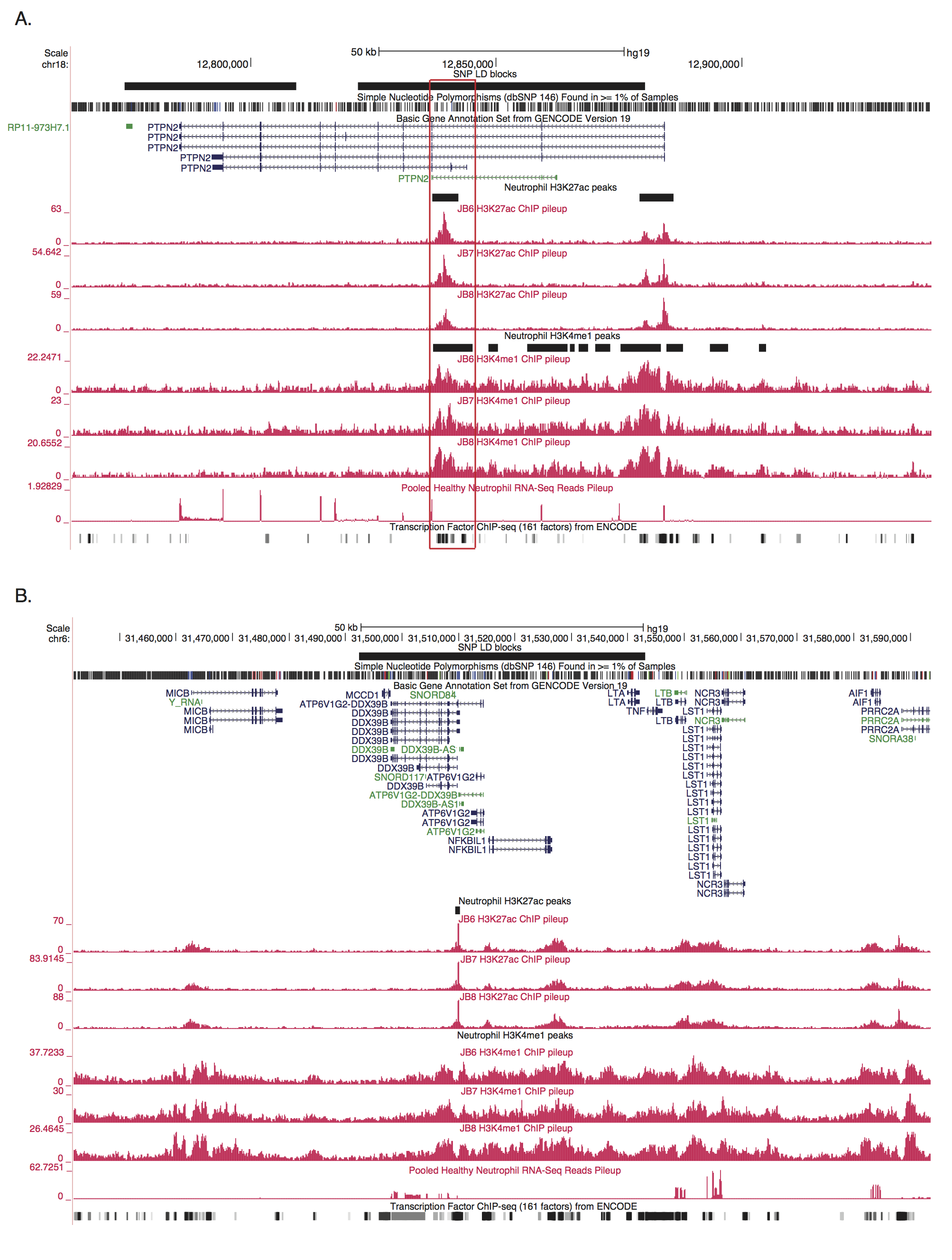

Supplement: Additional file 1: Figure S1. — Genome browser screen shots. Chromatin organization around PTPN2 and NFKBIL2 loci. (TIFF 20832 kb) [file 13075_2017_1260_MOESM1_ESM.tiff]

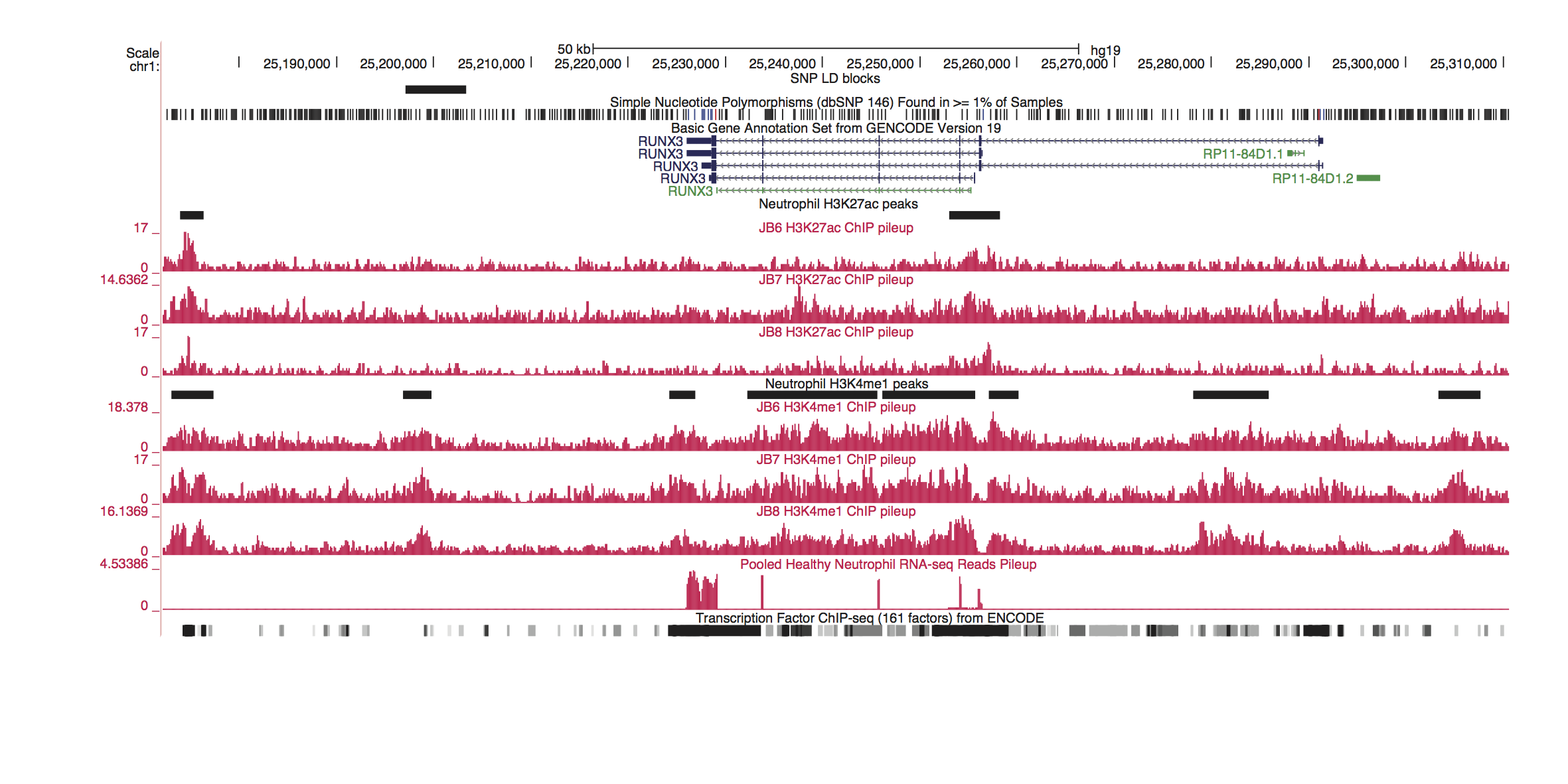

Supplement: Additional file 2: Figure S3. — Genome browser screen shots. Chromatin organization around the RUNX3 locus. (TIFF 10898 kb) [file 13075_2017_1260_MOESM2_ESM.tiff]

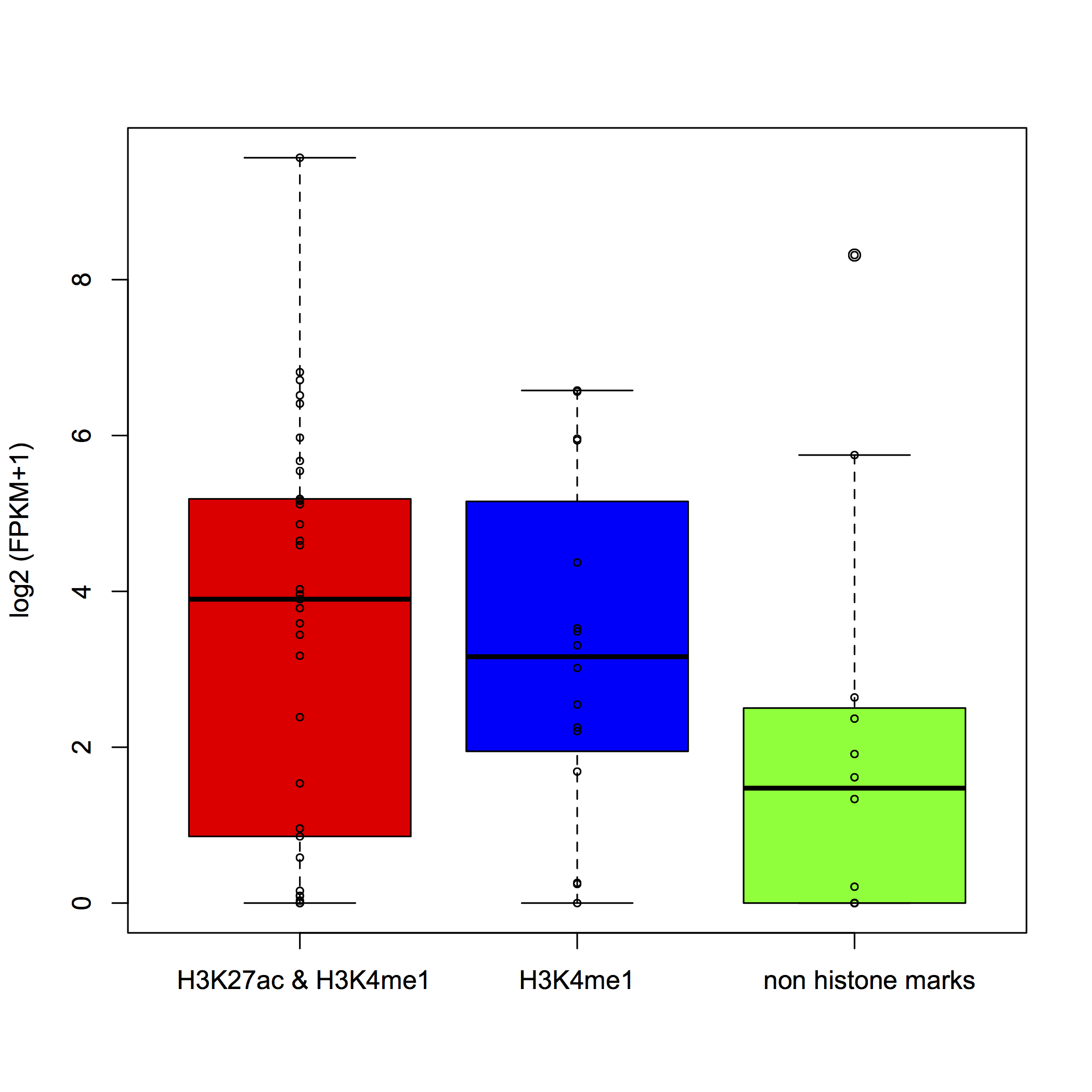

Supplement: Additional file 4: Figure S2. — Genes associated with these long-range interacting regions from CD4+ T cell ChIA-PET data. The genes associated with regions containing both H3K4me1 and H3K27ac marks have average higher FPKM than those genes that contained only H3K4me1 marks or those with no histone marks. (TIFF 17228 kb) [file 13075_2017_1260_MOESM4_ESM.tiff]
